# The Persian Brief Illness Perception Questionnaire: Validation in Patients with Chronic Nonspecific Low Back Pain

**DOI:** 10.1155/2021/3348011

**Published:** 2021-07-26

**Authors:** Sarvenaz Karimi-Ghasemabad, Behnam Akhbari, Ahmad Saeedi, Saeed Talebian Moghaddam, Noureddin Nakhostin Ansari

**Affiliations:** ^1^Department of Anatomy, School of Medicine, Guilan University of Medical Sciences, Rasht, Iran; ^2^Student Research Committee, University of Social Welfare and Rehabilitation Sciences, Tehran, Iran; ^3^Department of Physiotherapy, University of Social Welfare and Rehabilitation Sciences, Tehran, Iran; ^4^Department of Statistical Research and Information Technology, Institute for Research and Planning in Higher Education, Tehran, Iran; ^5^Department of Physiotherapy, School of Rehabilitation, Tehran University of Medical Sciences, Tehran, Iran; ^6^Research Center for War-Affected People, Tehran University of Medical Sciences, Tehran, Iran

## Abstract

**Background:**

Illness perceptions may influence coping behaviors as well as treatment and recovery among patients with chronic pain including low back pain (LBP). These perceptions may vary across different conditions. The Brief Illness Perception Questionnaire (BIPQ) is used as an instrument to assess the patients' perception of illness. Although the BIPQ has been previously translated into Persian, its psychometric properties have not been evaluated among patients with chronic nonspecific LBP. The aim of this study was to determine the reliability and validity of the Persian BIPQ in patients with chronic nonspecific LBP.

**Methods:**

116 patients with chronic nonspecific LBP with a mean (standard deviation) age of 36.4 years (10.7) participated in this cross-sectional study. Fifty patients were reexamined after 10 to 12 days for test-retest reliability. Internal consistency reliability, construct validity, concurrent criterion validity, and structural validity were evaluated. The concurrent validity was examined by using the Short Form-36 Health Survey.

**Results:**

There were no floor and ceiling effects. Cronbach's alpha for the total score was 0.90. The intraclass correlation coefficient (ICC) for test-retest reliability was 0.90. The standard error of measurement and the minimal detectable change was found to be 3.26 and 9.04, respectively. The convergent correlations confirmed the construct validity. The concurrent criterion validity was demonstrated by significant negative correlations with the SF-36. The Exploratory Factor Analysis produced the 2 factors (emotional illness representations and cognitive illness representations) with an eigenvalue >1.0 that jointly accounted for 58.86% of the total variance.

**Conclusion:**

The Persian BIPQ is a reliable and 2-factor instrument and can be used for assessing illness perception in patients with chronic nonspecific LBP.

## 1. Introduction

Low back pain (LBP) is one of the most common conditions associated with dysfunction, which has high social problems and financial costs [1, 2]. The LBP prevalence in Iran is estimated to be about 27% [3]. Epidemiological studies identified several psychological factors in addition to risk factors associated with work, which contribute to chronic nonspecific LBP [4].

It is recognized that the ways patients with various diseases perceive their illness and interpret their situation reflect their transient or chronic illnesses [5]. The paradigm of illness perception indicates how the patients view their condition in terms of cause, previous experiences, symptoms, expectations about the recovery process, and coping behaviors [6]. In the context of LBP, the illness perception of patients with LBP can influence their adaptation to their disease, the treatment efficacy, and LBP-related problems of pain and discomfort. In patients with chronic pain, negative illness perception has been shown to be associated with maladaptive behavior, dysfunction, and poor outcomes [7]. Since the illness perception of patients may have an impact on coping behaviors, the illness perception should be considered in the assessment and treatment planning of patients with LBP. It follows that the assessment of patients' perceptions of their LBP is important as their illness perception can influence their treatment outcome and recovery.

The Brief Illness Perception Questionnaire (BIPQ) is a simple, easy-to-used outcome measure to rapidly assess the impact of illness representations of cognition and emotion [8]. The BIPQ is a widely used, reliable, and valid measure applied in many countries. The BIPQ has been translated and culturally adapted into different languages [9–13]. The patients' illness presentation may vary across different patient populations, and that illness representation can affect coping behavior and the extent of complaints.

The BIPQ has been used and validated in people with various illnesses [14]. However, the Persian version of BIPQ has been only validated in diabetes populations and the psychometric properties are incompletely evaluated [13]. The authors suggest further validation studies with different languages versions [14]. The illness representation of patients with chronic nonspecific LBP and psychological perception of pain can affect the recovery course and risk of chronic complaints. It follows that the illness representation of patients with chronic nonspecific LBP should be determined and assessed using a reliable and valid scale. Therefore, the aim of the present study was to validate the Persian BIPQ in patients with chronic nonspecific LBP.

## 2. Methods

### 2.1. Design

This was a cross-sectional study to assess the psychometric characteristics of Persian BIPQ in patients with chronic nonspecific LBP. The study protocol was approved by the Ethics Committee at the University of Social Welfare and Rehabilitation Sciences (no. IR.USWR.REC.1396.205). All participants gave their written informed consent.

### 2.2. Participants

Persian-speaking patients who had at least 12-week LBP with no specific pathology were included. Participants were excluded if they had known pathology (e.g., neurological signs and symptoms, discopathy, spinal stenosis, history of psychiatric and psychotic diseases, cancer, infection, or any other conditions leading to the LBP). Patients with LBP referred to the university physiotherapy clinic by physicians who were eligible as assessed by an experienced physiotherapist were invited to participate in the study.

### 2.3. Sample Size

Guidelines suggest at least 100 subjects for the evaluation of psychometric properties in cultural adaptation studies [15]. In this study, we were able to include 116 subjects.

### 2.4. Procedure

A questionnaire was used to record the demographic data of sex, age, LBP duration, body mass index (BMI), and level of physical activity. After that, patients completed the Persian version of the BIPQ, Pain Catastrophizing Scale (PCS), Roland Morris Disability Questionnaire (RMDQ), Pain Anxiety Symptom Scale-20 (PASS-20), and Short Form-36 (SF-36) Health Survey. Fifty patients with stable BIPQ scores rated by the self-reported global rating of change scale recompleted the questionnaire after 10–12 days to evaluate test-retest reliability. The time interval between two administrations has been suggested as 1-2 weeks as appropriate for reproducibility evaluation [15].

#### 2.4.1. Brief Illness Perception Questionnaire (BIPQ)

The BIPQ has 8 items with 5 items on cognitive illness representations assessing consequences (Item 1), timeline (Item 2), personal control (Item 3), treatment control (Item 4), and identity (Item 5); 2 items on emotional representations assessing concern (Item 6) and emotions (Item 8); and 1 item that assesses illness comprehensibility (Item 7). The causal representation uses an open-ended response item asking patients to list the 3 most likely factors in having roles in their illness. Each item is scored on a 0 to 10 ordinal scale producing a total score from 0 to 80 and higher scores indicate higher negative illness representation of disease, chronic nonspecific LBP. The reliability and validity of the original English BIPQ have been demonstrated [8]. The Persian BIPQ was used in this study [13].

#### 2.4.2. Roland Morris Disability Questionnaire (RMDQ)

The RDMQ is a reliable and valid self-reported questionnaire on physical disability due to LPB [16, 17]. The RMDQ score ranges from 0 (no disability) to 24 (max. disability). The Persian RMDQ was used in this study [18].

#### 2.4.3. Visual Analog Scale (VAS)

The VAS is a self-reported measure of pain intensity. The patient is asked to represent the pain intensity by making a mark on the VAS line between the 2 of “no pain” and “ unbearable pain.” The VAS pain score is determined by measuring the distance on the 10 cm line between the “no pain” endpoint and the patient's mark [19].

#### 2.4.4. Pain Catastrophizing Scale (PCS)

The PCS is a widely used measure to quantify the types of thoughts and feelings of individuals when they are in pain. It is a 13-item scale asking patients about the degree of pain-related catastrophic thoughts using a Likert scale from 0 “not at all” to 4 “all the time.” The total score of PCS ranged from 0 to 52 [20]. In this study, the Persian version of PCS was used [21].

#### 2.4.5. Pain Anxiety Symptom Scale-20 (PASS-20)

The PASS-20 was used to measure pain-related anxiety. It consists of 20 items, each item is rated from 0 “never” to 5 “always” with the total score ranging between 0 and 100. A higher score indicates greater pain-related anxiety. The Persian version of PASS-20 was used in the present study [22].

#### 2.4.6. Short Form-36 (SF-36)

The eight-scale SF-36 is a quality of life widely used instrument that is used in different conditions including LBP to measure it in physical and mental aspects [23]. It has been validated in many foreign languages including the Persian language [24].

### 2.5. Data Analyses

The 2-way random-effects model intraclass correlation coefficient (ICC) with a 95% confidence interval (CI) was used to evaluate the test-retest reliability. The internal consistency was assessed by Cronbach's alpha. Values ≥ 0.7 are acceptable for ICC and Cronbach's alpha [15]. The standard error of measurement (SEM) (SD$\sqrt{1-\text{ICC}}$) and minimal detectable change (MDC) (1.96 √2.SEM) were used to evaluate absolute reliability. Pearson's correlation coefficient was used to assess the item-total correlation (ITC). Correlation coefficients ≥0.3 were considered acceptable [25]. To assess the construct validity and concurrent validity of the Persian BIPQ, Pearson's correlation coefficients were calculated and interpreted as excellent (1.0–0.81), very good (0.80–0.61), good (0.60–0.41), fair (0.40–0.21), and poor (0.20–0.00) [26]. The factor structure of the Persian BIPQ was analyzed using principal component analysis with varimax rotation. The cut-off point for factor loading was determined at 0.4 [27]. Floor and ceiling effects were calculated by computing the percentage of individuals who score lowest and highest possible score on the Persian BIPQ. The cut-off for significant floor and ceiling effects was set at 15%. The SPSS Statistical software version 17.0 (SPSS Inc., Chicago, IL) was used for all analyses.

## 3. Results

### 3.1. Characteristics of Participants

One hundred sixteen patients with LBP (82 females, mean age 36.4 ± 10.7 years, duration of LBP 28.3 ± 37.6 months; BMI 24.4 ± 3.6 kg/m^2^) participated in this study.

### 3.2. Floor and Ceiling Effects

Table 1 shows the mean and standard deviation for the Persian BIPQ and all outcome measures. The Persian BIPQ demonstrated no floor and ceiling effects; 2 (1.8%) had a minimum total score and 4 (3.6%) had a maximum total score. The total scores on the Persian BIPQ ranged from 19.0 to 68.0.

### 3.3. Internal Consistency Reliability

Cronbach's alpha for the Persian BIPQ was 0.90. The three items when omitted raised the alpha values; “Personal control, 0.912,” “Treatment control, 0.916,” and “Understanding, 0.902.” The alpha values when omitting an item, excluding those of above, were from 0.885 to 0.891.

The corrected item-total correlations for two items did not meet the cut-off value of 0.3 (personal control: −0.15, treatment control: 0.12).

### 3.4. Test-Retest Reliability

The test-retest reliability for the Persian BIPQ total score was excellent (ICC = 0.90, 95% CI: 0.82–0.94, *p* < 0.001).

### 3.5. SEM and MDC

The absolute reliability measures of the SEM and the MDC for Persian BIPQ were 3.26 and 9.04, respectively.

### 3.6. Construct Validity

The total score of the Persian BIPQ had a significant correlation with the total scores of the PCS (0.52, *p* < 0.001), RMDQ (0.51, *p* < 0.001), and PASS-20 (0.57, *p* < 0.001). There was no significant correlation between the Persian BIPQ and the VAS (*p* = −0.13).

### 3.7. Concurrent Validity

There were negative correlations between the Persian BIPQ total score and the SF-36 total score (−0.46, *p* < 0.001), SF-36 physical health (−0.44, *p* < 0.001), and SF-36 mental health (−0.40, *p* < 0.001).

### 3.8. Exploratory Factor Analysis

Results of the Kaiser–Meyer–Olkin (KMO) test (0.77) and the Bartlett test of sphericity (Chi-Square = 300.097, d*f* = 28, *p* < 0.001) showed the adequacy of sampling. Two factors were extracted with an eigenvalue >1.0 that together accounted for 58.86% of the total variance. The first factor (emotional illness representations, Items 1, 2, 5, 6, and 8; Cronbach's alpha = 0.929) accounted for 40.83% of variance (eigenvalue 3.27); the second factor (cognitive illness representations, Items 3, 4, and 7; Cronbach's alpha = 0.804) accounted for 18.03% of variance (eigenvalue 1.44) (Table 2).

## 4. Discussion

This study evaluated the psychometric properties of the Persian BIPQ in assessing the illness perceptions in patients with chronic nonspecific LBP and found it reliable and valid, in line with the original English version [8] and other language versions [11, 13, 14].

### 4.1. Floor and Ceiling Effects

There were no significant floor and ceiling effects in this study with chronic nonspecific LBP patients, indicating the content validity of Persian BIPQ. This finding further indicates that it is able to show the changes after interventions, either worsening or improvement. The floor and ceiling effects are not evaluated in patients with type I diabetes that used the Persian BIPQ [13]. The floor and ceiling effects are not evaluated for the original English version [8] and other language versions of Chinese, Polish, Dutch, and Malay [9–11]. Floor and ceiling effects were not reported for the Malay BIPQ total scores [12]. A further study with the Dutch version of the BIPQ in acute nonspecific LBP patients did not evaluate the floor and ceiling effects, as well [28].

### 4.2. Internal Consistency Reliability

The Cronbach alpha for the total score found in the present study exceeded the cut-off value of 0.7. The alpha values if each item was omitted were also satisfactory and did not change substantially relative to the alpha of the Persian BIPQ total score. These findings indicate the all items in the Persian BIPQ are necessary for measuring illness perception, consistent with those reported for the versions of Dutch (Cronbach's alpha of 0.73) [28], Chinese (Cronbach's alpha of 0.783) [9], Polish (Cronbach's alpha of 0.74) [10], and Turkish (Cronbach's alpha for subscales between 0.715 and 0.774) [29]. The internal consistency reliability is not evaluated for the versions of original English [8], Malay [12], Dutch in patients with Chronic Obstructive Pulmonary Disease (COPD) [11] and the Persian BIPQ used in patients with diabetes [13].

### 4.3. Test-Retest Reliability

In this study, the ICC for the Persian BIPQ total score showed excellent test-retest reliability which was better than those reported for the original English BIPQ [8] and the Persian BIPQ evaluated in patients with diabetes [13]. The higher test-retest reliability in our study with Persian BIPQ (ICC = 0.90) might be explained by the fact that the ICC was calculated for the total score while the correlation values for the original English BIPQ (range 0.48–0.70 with a 3-week interval, range 0.42–0.75 with a 6-week interval) [8] and those reported with Persian BIPQ in diabetes patients were calculated for the items. The translated versions of Dutch (ICC = 0.72) [28] and Malay (0.39 to 0.70 with a 2-week interval, and 0.58 to 0.78 with a 4-week interval) [12] found acceptable test-retest reliability as well. The Dutch version evaluated in patients with COPD found test-retest reliability at 1 week, weighted Kappa >0.70 for the consequences, concern, and emotional response, and weighted Kappa <0.70 for the personal control, treatment control, and identity [11]. Test-retest reliability for the translated versions of Polish [10] and Turkish [29] is not evaluated. The excellent test-retest reliability found in this study for the Persian BIPQ indicates the stability of the measurements in patients with chronic nonspecific LBP.

### 4.4. SEM and MDC

The SEM and MDC were calculated in this study as the measures of absolute reliability. They are important measures as they provide information helping clinicians to be ensured that the change observed after treatment is real and not an error in the measurements. The SEM calculated in this study is small that indicates the reliability and sensitivity of the Persian BIPQ.

The MDC is important as it defines the minimal difference between measurements required to be considered real for a change in the Persian BIPQ scores. The MDC for the Persian BIPQ was 9.04; this indicates that changes in scores achieved by a patient with chronic nonspecific LBP must be ≥ 9.0 point to be defined as a real change that occurred due to an intervention. The MDC was reported for the Dutch language version (MDC_individual_ 3.0–4.0, MDC_group_ 1.0) [11]. Differences in the MDC values could be that the MDC was calculated for the Persian BIPQ total score, while the MDC was calculated for each dimension of the Dutch language version. Another possible reason for the variability between the 2 studies could be from the population included as the SEM and MDC vary with the population from whom these values are calculated, chronic LBP in this study and COPD in the study of de Raaij et al. 2012 [11]. A study with patients with acute nonspecific LBP used the Dutch version and reported a MDC of 42 points [28] that is much larger than that we found in our study. The larger MDC value reported for the Dutch version could be due to either the acuteness of the LBP patients, random error in the Dutch BIPQ scores, or low agreement between the test and retest scores [28]. The SEM and MDC are not evaluated for the original English [8] and the other adapted versions [9, 10, 12, 13, 29].

### 4.5. Construct Validity

To assess the construct validity of the Persian BIPQ, various measures were used. The Persian BIPQ, as hypothesized, showed significantly good positive correlations with the RMDQ, PCS, and PASS-20, while no correlation was detected between the Persian BIPQ and the VAS pain. This finding indicates the construct validity of the Persian BIPQ and the influence of the functional and psychological aspects of chronic LBP on illness perception in this population. In other words, the functional and psychological consequences of pain are important in the determination of illness perception in patients with chronic LBP [30]. It follows that emotional factors such as anxiety and beliefs about pain catastrophizing are associated, which in turn can influence the illness perception and development of functional disability. The implication of this finding is that the Persian BIPQ measures a construct covering the functional and psychological aspects of low back-related pain and illness perception or mental representations and personal ideas about the illness which indicates the usefulness of Persian BIPQ [14]. The construct validity of the Persian BIPQ is consistent with that reported for the Chinese and Malay versions [9, 12].

### 4.6. Concurrent Validity

To examine the concurrent validity of the Persian BIPQ, we assessed the correlations between the Persian BIPQ and the SF-36. As hypothesized, we found good negative correlations between the Persian BIPQ total score and the SF-36 total score and the SF-36 subscales of mental health and physical health. The significant negative correlations between the two indicate the influence of illness perception of patients with chronic LBP included in this study on the quality of life such that the negative illness perception of individuals about their pain and disability was associated with the low quality of life. Our findings are consistent with the original English version that used the mental health subscale of the SF-36 to determine concurrent validity in patients with myocardial infarction and found negative correlations for 4 items of the BIPQ [8]. These findings support the concurrent validity of the Persian BIPQ in agreement with the original English and translated versions [8, 10, 11, 13, 28]. A systematic review and meta-analysis of the BIPQ with 188 papers administered in various illnesses from 26 languages and 36 countries demonstrated the concurrent validity of the BIPQ [14].

### 4.7. Exploratory Factor Analysis

In this study, the dimensionality of the Persian BIPQ was assessed by examining the ITC and the exploratory factor analysis. The 3 items of “Personal control,” “Treatment control,” and “Understanding” when removed relatively raised the alpha values. This finding together with the low ITC values for the items “Personal control” and “Treatment control” indicates that the Persian BIPQ is not a one-dimensional instrument. These 3 items constitute a factor as confirmed with the factor analysis (cognitive illness representations, Items 3, 4, 7). The understand item is a meta-cognition item and it seems logical to be included with the items defining the cognitive representation of illness. It follows that the remaining 5 items also extracted from the factor analysis constitute the second factor (emotional illness representations, Items 1, 2, 5, 6, 8). The ITCs are not reported for the original English BIPQ [8] and while the factor analysis is not performed, the dimensionality of the BIPQ (cognitive illness representations and emotional illness representations) has been acknowledged [14]. Our 2-factor solution for the Persian BIPQ is in agreement with the original English and translated versions [14] supporting its internal structural validity.

The two factors produced for the Persian BIPQ in the present study are exactly similar to those found for the Turkish version using a confirmatory factor analysis [31]. The Arabic version of BIPQ used in a group of patients with cardiac disease reported corrected ITC between 0.220 and 0.588; the factor analysis was not performed for the Arabic version [32]. The validation study of the Chinese version of BIPQ in Hong Kong Chinese breast cancer survivors reported a 2-factor solution (cognitive illness representations subscale and emotional illness representations subscale) with the understand item omitted [9]. The factorial validity was not evaluated with the Persian BIPQ in diabetes patients [13], Malay [12], Polish language version [10], and Dutch [11, 28].

### 4.8. Limitations

The limitations in this study must be noted. First, this study adopted a cross-sectional design. A further study considering a longitudinal study design is required regarding predictive validity and changes in illness perceptions over time. Second, the discriminant validity and predictive validity were not evaluated. Third, although the lack of floor and ceiling effects imply the responsiveness of Persian BIPQ [33], responsiveness was not evaluated in this study in the context of an intervention trial using effect size methodology. Forth, this study treated the Persian BIPQ as a single construct tool and used the total score to reflect that all items measure the same illness perception construct. However, the BIPQ is an instrument with single-item scales and it thus requires the items to be individually analyzed. A study to compare the Persian BIPQ total score relative to the individual single-item score is warranted to establish which scoring approach is appropriate for the interpretation of illness perception. Fifth, the causal dimension of Persian BIPQ was not evaluated in this study.

## 5. Conclusion

The Persian BIPQ demonstrated excellent reliability and validity as a 2-factor questionnaire for assessing the illness perception in patients with chronic nonspecific LBP. Further examination of Persian BIPQ in a longitudinal study is required to assess the predictive validity, discriminant validity, responsiveness, and changes in LBP perception over time.

## Figures and Tables

**Table 1:** Descriptive statistics of the Brief Illness Perception Questionnaire (BIPQ) and other scales.

	Mean	SD	Minimum	Maximum
VAS	4.2	1.7	0.0	10.0
PCS	13.7	8.0	0.0	36.0
RMDQ	7.1	4.4	0.0	20.0
PASS-20	35.6	18.4	0.0	90.0
SF-36-PH	59.5	19.0	14.3	96.0
SF-36-MH	58.8	20.3	5.4	100.0
SF-36	59.1	17.9	13.4	98.0
BIPQ	44.2	10.3	19.0	68.0

VAS: visual analog scale; PCS: pain catastrophizing scale; RMDQ: Roland Morris disability questionnaire; PASS-20: pain anxiety symptom scale-20; SF-36: short form-36; SF-36-PH: short form-36-physical health; SF-36-MH: short form-36-mental health.

**Table 2:** The factors of Persian BIPQ^*∗*^.

Persian BIPQ items	Factor 1	Factor 2
	Emotional illness representations	Cognitive illness representations
	Factor load	Factor load
Consequence	0.874	
Timeline	0.700	
Identity	0.738	
Concern	0.822	
Emotional response	0.781	
Personal control		0.582
Treatment control		0.724
Understanding		0.730

^*∗*^Brief Illness Perception Questionnaire.

## Data Availability

The datasets used and/or analyzed in the current study are available from the corresponding author on reasonable request.
